# Dental Neuralgia

**Published:** 1851-01

**Authors:** A. C. Dayton

**Affiliations:** Dentist, Vicksburg, Miss.


AMERICAN JOURNAL

OF

### DENTAL SCIENCE.

Vol. I. NEW SERIES—JANUARY, 1851. No. 2.

### ARTICLE I.

*Dental Neuralgia.* By A. C. DAYTON, M. D., Dentist, Vicksburg, Miss.

THE sensitive nerves of every part of the body are liable to be affected by that peculiarly distressing and painful disease, commonly called neuralgia or tic douloureux. If all the nerves were equally exposed to its exciting causes, it is probable we should meet with it as often in one part as another. This, however, is not the case. It affects the nerves of the head and face almost exclusively. We find it oftener in some of the branches of the fifth pair of nerves than in all the other nerves of the whole body. So true is this, that systematic writers on this disease for a long time recognised and described only four varieties, (see Cooper's Surgical Dictionary,) which they call, 1st. Frontal neuralgia, the seat of which is that branch of the fifth pair which comes out above the socket of the eye, and is spread upon the forehead. 2d. Suborbitary neuralgia, affecting that branch of the fifth pair which comes out below the eye, and is spread upon the cheek by the side of the nose. 3d. Maxillary neuralgia, affecting the nerves of the lower jaw, chin and lips; and 4th. Facial neuralgia, which, it is said, has its seat in those branches of the seventh pair which are spread upon the face, but which are so intimately connected with the facial branches of the fifth pair, that it is hard to tell in which it commenced, as both are early involved in the disease, and their fibres are so interlaced and intermixed, as to render any distinction difficult, if not impossible.

This painful affection sometimes, however, occurs in other nerves. Denmark mentions a case involving the nerves of the arm. Abernethy speaks of a case affecting one of the fingers. Sir Astley Cooper and other writers mention similar cases, but they are so rare and so irregular, that they have not been classified and systematically described in the text books, while those affecting the several branches of the fifth pair of nerves, are of constant and regular occurrence.

From the above facts, it is evident that these nerves are subject to the influence of some cause of disease, which does not act with equal force on the other nerves.

This cause is dental irritation. The fifth pair of nerves supplies the teeth of both the upper and lower jaws. The terminal dental branches are exposed to many sources of irritation from which the other nerves of the body are usually free, such as the contact of the air—of the fluids of the mouth—and of alimentary substances; the influence of dead roots of teeth; pressure from the enlargement of the ends of the roots of teeth, inflammation of the gums, inflammation and suppuration of the sockets, &c., &c.

Neuralgia originating from this cause, may very properly be termed DENTAL NEURALGIA; and the object of this paper is to investigate the pathology and treatment of the disease.

There is no fact, in the history of disease, better known, or more universally admitted, than that the *suffering* produced by it is often felt in a part distant from that which is the actual seat of the disorder; the *seat* of irritation is in one organ, the pain, and often other apparent *effects* of the irritation, are manifested in another. Thus, inflammation of the stomach causes pain in the head; inflammation of the liver is felt in the shoulder; disease of the hip joint causes pain on the inside of the knee; irritation in the bowels sometimes causes cough, pain in the chest, difficulty of breathing; and, indeed, nearly all the symptoms of inflammation of the lungs, or, in other cases, of inflammation of the brain.

I doubt if we are yet in possession of such information as will enable us perfectly to understand and explain these phenomena. It is common to say, that they occur from the influence of *sympathy.* We say that the shoulder *sympathises* with the liver; that the head sympathises with the stomach, the knee with the hip, and the lungs and the brain with the intestinal tube. I use the word as others have, and will proceed to examine some of the extensive and wonderful sympathies which are connected with the teeth and their appendages.

Let us see if we cannot attain to at least a partial understanding of these mysterious influences. The careful and attentive observer of disease cannot fail to discover that they most frequently exist between organs which are supplied by branches of the same nerve, or by nerves which have their origin near each other in the brain, and it seems most probable, that the diseased influence is transmitted from the organ first affected, through its proper nerve, to the brain, where it causes irritation at the roots of the nerve through which it was sent, and this irritation extends to the contiguous portions of the brain, and is thence sent back through the other branches of the same nerve, or through the other nerves which have their origin in the affected region, so as to be manifested or felt in the organs on which they are spread, or in the fibres of the nerves themselves. If this explanation is the true one, (and it harmonizes so well with all the facts, that I cannot doubt its truth,) it will enable us to understand, in part at least, the influence of the diseases of the teeth, not only in causing neuralgia of the head and face, the eye and ear, and other parts supplied by branches of the fifth pair and their own nerves, but of other parts which are associated with them only by deriving their nerves from the same locality in the brain.

In most cases of dental neuralgia, however, the pain is felt in another branch of the fifth pair, as we have already had occasion to say. The seat of irritation is in the branches supplying the teeth, while the pain is felt in branches supplying the temple, the forehead or the face, the ear or the eye.

In order to understand this more perfectly, let us go a little into detail. A tooth in the lower jaw is decayed, and its pulp, containing the terminal extremity of its nerve, exposed. The pain is at first felt in the tooth. It is simply tooth-ache. The irritation very naturally extends down through the root to the main trunk of the submaxillary nerve. It then seems to the patient to involve all the teeth on that side of the jaw. He can hardly say which aches most, or which was the original seat of the disease. He now calls it "jaw-ache." By further extension, the branches supplying the chin and lip are implicated. In time the disease extends back, and involves other branches, which come off nearer the brain. Then the pain is felt in the temples and on the side of the head. In the mean time, the pain in the tooth has ceased. The patient, occupied with his more intense and more extensive sufferings, perhaps forgets that it was ever painful. The pain may continue to affect the whole course of the nerve and its branches, or it may locate at some point and become an independent disease. Such is the history of many cases of "maxillary neuralgia," as it is commonly called.

An upper tooth may be affected in the same way. It receives its nerves from the upper maxillary branch of the fifth pair. The irritation extends to the main trunk of the nerve. Some branches of it supply the eye, which may become the seat of pain or of more serious disease, such as extreme sensitiveness to light, dimness of vision, and, as has happened in some cases, of absolute blindness.

A branch of this nerve comes out above the eye, to be spread upon the forehead. When this becomes the seat of pain, it is the frontal neuralgia of the authors.

Another branch of this nerve comes out below the eye, and is spread on the cheek by the side of the nose, and when this becomes the seat of pain, it is the suborbital neuralgia.

The filaments of these nerves, and those of the seventh pair are so intermixed and interwoven in the face, that one is liable to be affected by the other; and if the seventh pair becomes affected in this way, we have what is described as facial neuralgia.

But if we examine no further than this, we will have but a very imperfect conception of the matter. This is the most *direct*, but probably not the most *common* mode in which the diseased condition of the mouth produces neuralgia. It operates, also, indirectly through the stomach, through the brain, or through the general nervous system.

1st. *Through the stomach.*—Irregularities and errors in diet have long been reckoned among the active causes of neuralgia. They of course produce their first effect upon the stomach. The nervous disease is a consequence of the stomach's derangement. The pain in the head or face is sympathetic, and may be caused as well by *any thing else* that will derange the action of the stomach, as by an error in diet; and it is a well known fact, that the diseased condition of the mouth is a common cause of disease in the stomach.

But this is not all: persons who are habitually dyspeptic are more liable to neuralgia than others. The general debility of the system, and the irritable condition of the nerves, which disease of the stomach, sooner or later, is sure to produce, strongly predispose the patient to nervous disease, and it requires only some local irritant to concentrate this increased irritability at some particular point, in order to develop the most tormenting neuralgia.

Thus it is evident, that the diseased condition of the mouth may act in a *double* capacity in the production of these pains. It first produces dyspepsia, which induces a strong predisposition to them, and then furnishes the local irritant to call them into action.

2d. *Through the brain and general nervous system.*—The influence of great mental excitement from close application to study. Unusual anxiety or depression of spirits in the production of neuralgia has been generally recognised by medical

10*

writers. These acting directly on, and through the brain, occasion the disease of the nerves.

It has been observed, also, that those who are subject to hysteria, in any of its various forms, or to that vague and undefined disease or state of the system, commonly called "nervousness," are from the great irritability of the whole nervous system peculiarly subject to this disease.

If, therefore, the diseased condition of the teeth and mouth is likely to affect the brain and general nervous system, so as to produce this state of things, it is easy to understand another of the channels through which neuralgia is produced. And no one who will recollect the influence of the pain of tooth-ache, of the loss of sleep, of the deranged digestion, and the opiates and other abominable drugs which are put in the mouth to relieve the pain, will, for a moment, doubt that both brain and nerves are thus affected by the diseased condition of the teeth.

In view of all these facts, it does not seem strange to me that the fifth pair of nerves in some of its branches is more frequently the seat of neuralgia than all the other nerves combined.

Dental irritation is often both the predisposing and the exciting cause, and even when the *predisposition* arises from some *other* source—as is often the case—the local irritant which excites and determines the position of the pain is found in the mouth.

But dental neuralgia is by no means confined to the fifth pair of nerves and its branches. The condition of the mouth is sometimes such as to produce that state of the nervous system most favorable to the development of the disease; while for the same reason the pain is located in a distant part.

Dr. Fuller says, "He has twice extracted teeth when the most severe pain was in the elbow. In both instances it was one of the large grinders in the lower jaw; and in both the pain in the elbow vanished on removing the teeth." Dr. Koecker (if I remember right) mentions a similar case.

Dr. Bell says, "It not unfrequently happens that parts the most remote become the apparent seat of pain, from the exposure of the nerves of a tooth. I have seen this occur, not only in the face, over the scalp, in the ear, or underneath the lower jaw, but down the neck, over the shoulder, and along the whole length of the arm."

Dr. Rush mentions a case of rheumatism in the hip, (which was, I have no doubt, neuralgic pain,) which he cured by the extraction of a diseased tooth.

Dr. Fitch describes a case in which the pain extended down the arm, and even to the ends of the fingers.

Sometime during the summer of 1846, I was requested by her physician to see Mrs. R——, of Tennessee. She was about forty-five years of age, and had in early life possessed a fine constitution. For some ten years, however, she had suffered from what some of her physicians had called rheumatic pains, affecting the right side of the neck—the right arm, and at one time, the whole of the right side of the body. The physician who was in attendance at the time I speak of regarded it as a case of neuralgia, caused by the pressure of a large goiter, which disfigured her neck upon the cervical nerves, until while listening to one of my lectures he was struck with the probability of its being a case of dental neuralgia, and immediately desired my opinion and advice.

When I saw her I learned that she could not get out of the bed without assistance—she had a distressing cough—suffered greatly from dyspepsia, was greatly emaciated, and had been confined to the house much of the time to her bed for five years.

With the assistance of two persons I got her placed in a chair, and bolstered about with pillows to support her while I could make an examination of her mouth. I found her teeth in a dreadful condition. In two, the pulp was exposed, some were dead, though little decayed, others had lost their crowns. The gums were greatly inflamed. The sockets of nearly all the teeth were suppurating, some of the teeth were loose and all were more or less covered with tartar.

I assured her that I had no doubt of being able to relieve at least the most distressing symptoms of her disease, and that I might probably effect a permanent cure if she would allow me to restore her mouth to a healthy condition.

She promptly consented to this, and I extracted four teeth before removing her from the chair. She then appeared to be exhausted, and was replaced on her bed. About two hours afterwards I had her placed in the chair again, and extracted four more.

The next day I found her free from pain and stronger. She was able to sit up while I removed the tartar from about her remaining teeth. In three days she was able to walk without assistance into another room, when I extracted several more. I directed the use of a gently stimulating and astringent wash, and promised to see her again in two weeks and complete as far as practicable, what was to be done for her mouth.

Feeling anxious about the case, however, I called in about ten days to see how it was progressing. To my surprise, I learned that she considered herself quite well, and had gone fifteen miles into the country to visit a relation.

In consequence of this imprudence, she suffered a slight relapse, but in a few months, not only had the pains in her neck, side and arm entirely disappeared, but her general health and strength was almost wholly restored. I met with her physician last winter, and he informed me that she still continues well and attends, without inconvenience, to the cares of a very large family.

### *Diagnosis.*

How is dental neuralgia to be distinguished from neuralgia which has its origin in general causes, or in other local irritation? It is not easy to answer this question by any definite or invariable rule.

*The locality of the pain is not a distinguishing sign;* for not only is neuralgia of the head and face, but also of the neck, side, hip and arm, (as we have seen,) sometimes dependent on the teeth, and more than this, it occasionally happens that the pain is felt in the teeth themselves, when the cause is in some other part.

A young gentleman applied to me in 1837, to extract one of his lower large grinders, which he said was the seat of the most tormenting tooth-ache. I made a careful examination of the tooth, and found in it no sign of disease. Indeed, *all* his teeth in both jaws were perfectly sound and remarkably beautiful. There was not the slightest evidence of disease in any part of the mouth except this pain.

On inquiry, I learned that he had been subject to chills and fever for several weeks previous to the attack of tooth-ache, and that the paroxysm of tooth-ache came on every day about the same time, at which he used to have the chill. Here was evidently a case of neuralgia of the *dental nerves* from a general cause. It would have been worse than folly to sacrifice the teeth. I ordered a cathartic to be taken that night, and full doses of quinine the following day. On the second day afterwards I had the satisfaction to learn that his tooth-ache was cured. It did not return, and he had still his mouth full of beautiful teeth, which might all have been extracted without relief to the pain.

I know of a case of neuralgia affecting the abdominal viscera from a similar cause. It had been mistaken for bilious colic by the patient and his medical attendant. It came on regularly every fourteen days for several months, and its regularity of return did not attract the attention of any one until in looking over his account in the doctor's book one day, I chanced to observe the peculiar regularity in the intervals of his visits, and suggested to him that it might be a case of miasmatic disease. He was struck with the idea, and immediately visited the patient and put him on the use of quinine. I learned from him afterwards, that he had no more of the "colic."

As the irritation of the dental nerves is often reflected upon the other branches of the fifth pair, so irritation of other branches is sometimes reflected back upon the teeth :

Dr. John McCalla, in the Journal for July, 1848, relates a case which furnishes an admirable illustration of this truth.

Miss S. D. had been subject to neuralgia in the lower jaw for two years. Having some defective teeth on the painful side of her face, they were removed with temporary relief. She was subsequently treated with tonics, and among others, she took arsenic, which lessened the duration but not the regularity of the pain.

On combing her hair she discovered a tender spot, which, when pressed rather sharply, gave her one of the severest attacks of pain in the jaw. On examination, a tumor was found about the size of a horse bean, situated on the parietal bone. Pressure was again repeatedly tried and always produced the same severe pain. The tumor was removed with permanent relief and speedy return to health.

Dr. Darwin says, in his Zoonomia, that whenever he has found neuralgia on the side of the head, confined to a spot near the centre of the parietal bone, it has always been connected with disease of the lower grinders. If this be a general rule, it will afford a valuable sign in the few cases in which the pain is confined to that locality. These, however, make but a very small part of the whole.

### *Periodicity of the Paroxysms is not a Distinguishing Sign.*

It has commonly been taught, that in those cases which depend on *general* causes, the paroxysms of pain are regularly periodical, while, in those dependant on the teeth, or other *local* causes, they return at irregular intervals or are almost continuous.

This distinction is made, I think, on the authority of Mr. Bell, and has been commonly recognized by other writers. I have not found it to be true. In my experience this sign has proved of little or no value.

In 1847, Mrs. ——— of Adams county, Miss., called at my office. She had suffered for some five months with a most excruciating pain which occupied a spot about an inch in diameter in the right temple. *It returned regularly every night about nine o'clock*, and continued about two hours, when it gradually wore off and she was able to sleep. She was then free from pain until the *same time* next day. Her general health was tolerably good, though she was slightly dyspeptic, and suffered somewhat from "nervousness."

As the pain had been *regularly periodical*, her medical advisers, (and she had consulted several of the most eminent physicians in the state,) considered it as having a constitutional origin, and had prescribed for her a variety of remedies which she had taken without the slightest permanent benefit.

Most of her teeth were sound and apparently healthy. She had, however, lost the crown of one and suspecting the remaining root as the cause of her suffering, she desired to have it extracted. This was accordingly done, and I expressed a hope that she might not suffer any more, but desired her, if the paroxysm should return as usual, to call again and let me examine her mouth more carefully.

She called again after three days and informed me that she discovered no change, either in the time or severity of the attacks of pain. I placed her in proper position in my operating chair, and with the necessary mirrors and instruments commenced a careful and thorough examination of every part of her mouth, tooth by tooth. I found no decay or other disease of any consequence in the lower jaw, but, in examining the upper teeth, I found one which was deeply decayed very near to the gum. When the point of the instrument passed into the cavity and touched the exposed nerve, she sprang up and placed her hand on the side of her head, exclaiming, "there is the pain now." She did not even then feel it so much in the tooth as in the temple.

It was on some account peculiarly desirable to preserve this tooth, and I proposed to make an effort to do so, informing my patient that I hardly hoped for success; but if I failed, she would only lose the tooth after I had tried.

I put in the cavity a preparation to destroy the vitality of the pulp, and desired her to call again in three days. She did so, and informed me that she had no pain that night, nor afterwards. At a proper time I plugged the tooth with gold. I saw her again more than a year after this operation. The tooth was still sound, healthy and useful, *and she had never had the slightest return of the neuralgia in the temple.*

John Hunter relates the following case, in which the parox ysms were regularly periodical, yet dental irritation was certainly the cause of the pain.

A lady about twenty-six years of age, was attacked with a violent pain in the left side of the face, *it was regularly periodical, coming on about six o'clock every evening.* She took Peruvian bark, which had no effect, she took antimonials and Dover powders, which were equally ineffectual. But one of the points of the wisdom tooth of the upper jaw coming through, showed the cause, and indicated the remedy. The gums were lanced and the pain ceased.

Dr. Darwin says, "A remarkable circumstance attends this kind of hemicrania, viz. that it recurs by *periods* like those of intermittent fever, *and that even when a decaying tooth is the cause* as is evinced by the cure of the disease by the extraction of the tooth.

It will be found in practice, that cases which are at first irregular, frequently become periodical while those which were at first periodical, sometimes (though more rarely) cease to be so. It is well known that in miasmatic districts there is a tendency in all chronic diseases to assume a periodic type, and it is probable that this circumstance may explain the periodic character of many cases of neuralgia, let the original cause have been what it may.

The following case from Mr. James Robinson's admirable work on the teeth is so perfectly coincident with what I have frequently observed, that I cannot forbear to quote it:

"We were consulted by a lady about twenty two years of age, who for eight months had suffered pain in the branches of the maxillary nerves, superior and inferior, which pain *at first* came on in irregular paroxysms, but had latterly become *distinctly periodical*, invariably commencing at nine o'clock, A. M., and seven, P. M. The severe character of the attacks generally lasted about an hour. They seldom occurred at any other time than those specified, unless the patient was suffering from indisposition or mental disquietude.

Early during her sufferings, she was persuaded to apply salt and brandy, with mustard poultices, &c. but these had no good effect.

She then consulted an eminent physician, who ordered various preparations of iron, combined with quinine, which medicines were continued for two months without the slightest relief.

Belladonna was next tried, commencing with a grain, night and morning, an hour before the paroxysms came on. Leeches and blisters were applied to the temples, and fomentations of poppies and chamomile, and the belladonna was increased to three grains, twice a day.

She now became so much affected with vertigo and dimness of sight, [the effect of the belladonna,] that this treatment was discontinued, and iodide of potassium was substituted, with the internal use of veratrine.

These measures were pursued unsuccessfully for six weeks, when it was suggested by a medical friend, that the disease *possibly originated with a diseased tooth*, and under this suggestion we were consulted.

On examination, it was found that all the grinders in the lower jaw, and two in the upper jaw, were carious. It was very evident that this mass of caries was producing great irritation in the surrounding structures. When the diseased teeth were sounded with a steel instrument, the paroxysm of pain occurred with its usual violence.

We immediately removed the diseased teeth from the lower jaw, and ordered the following: acetate of morphia, one-fourth of a grain; camphor mixture, half an ounce; to be taken as a draught. Also the following aperient: extract of colycinth, six grains; calomel, two grains; to be taken at bed-time.

These measures produced considerable relief, and in the course of a week she had the other diseased teeth removed.

A few months since, we had the satisfaction of hearing that she was quite recovered, and *had not experienced a moment's uneasiness in the jaw since the last operation.*

From such cases as these, to which I might add several more from my own observations, I am satisfied that the periodicity or non-periodicity of the poroxysms is not a sign which can be at all relied upon, to distinguish dental neuralgia from neuralgia originating in other sources; for, although it is undoubtedly true, that those few cases that originate entirely from the influence of marsh miasma are distinctly periodical, yet it is also true, that neuralgia from dental irritation is, in many cases, equally regular in the recurrence of the paroxysms of pain.

I have dwelt longer upon this point than I should have done, but for my anxiety to set aside a rule in practice which is not sustained by facts, and the adoption of which has led to so much malpractice.

How, then, shall we distinguish these cases? Something may be learned from the influence of remedies. If the disease have originated from the influence of miasma, it will be probably cured by the proper use of tonics. If it have arisen from some general disease of the nervous system, or some disorder of the liver or stomach or brain, it will probably be relieved by remedies addressed to these organs. But it must not be forgotten that the disease of the stomach, liver, spine or brain may itself depend on the condition of the mouth; and the restoration of the teeth and gums to health, may consequently be as necessary to the final and perfect cure of the neuralgia, as though dental irritation had been *more directly* the cause. And it is important to remember, also, that these general diseases often produce only predisposition to the neuralgia, by causing an irritable and excitable condition of the nervous system, which the dental irritation concentrates and develops into an active neuralgia. And, therefore, although we may see the apparent cause of the disorder in these general derangements, it does not follow that the diseased condition of the mouth is not also concerned in them.

The following case will illustrate my meaning:

Mrs. B——, aged about thirty, of delicate constitution and nervous temperament, in consequence of great mental excitement during her pregnancy, became subject to an affection of the uterus, which caused the loss of her general health. She became subjeet to convulsions, almost epileptic in their character, and a variety of nervous affections. She was, when I first saw her, suffering from dyspepsia, and chronic disease of the liver. Her mind was mnch impaired, her eyesight dim, her skin sallow and parchment-like, and her whole appearance indicated a very great derangement of the vital functions.

The whole right side of the head and face was subject to the most severe attacks of neuralgia. The pain was sudden and sharp, shooting along the nerves, and often attended by spasms of the muscles on that side of the face.

The uterine affection was at length cured, or so greatly relieved as to cause her but little trouble, and a very great improvement in her general health was the consequence. The neuralgia, however, still continued. It was not quite so severe, but the attacks were as frequent as before. A great variety of remedies had been employed for it, without affording the slightest permanent relief.

By advice of her physician, she had applied to a dentist to restore her mouth to health. He operated extensively upon her teeth, (which were very bad,) but she was rendered much worse by his interference, and had consequently given up all hope from this source.

Some time afterwards she was very reluctantly induced, by the earnest request of her physician and some of her friends, who had derived advantage from my services, to place herself under my care. When I first saw her, I was not allowed to examine her mouth. When I did so, I found it in a most dreadful condition. There were only four or five teeth in each jaw that could be preserved at all. Her gums were in a state of obstinate inflammation, which had evidently been much increased by the previous dental treatment, by which several teeth had been cut off or broken, and the roots left in the jaw.

I extracted twelve teeth and roots, and directed proper applications to the diseased gums. By this treatment, the paroxysms of pain were, in a few days, greatly diminished, both in severity and frequency; but shooting pains and slight spasms continued for several months, until after artificial teeth had been inserted to supply the place of those she had lost, and enable her to masticate her food.

*Then, as her stomach gradually resumed its healthful action, her nervous system acquired new vigor, and the neuralgia, with the spasms of the face, entirely disappeared.*

In this case, it is probable that the *original* cause of the neuralgia was the uterine disturbance; but when once the nervous system had become debilitated, the diseased condition of the teeth, and consequent derangement of the functions of the stomach and liver, were sufficient to keep up the disorder and prevent the cure of the disease, even though the original cause of it had been removed.

The general rule of diagnosis, which in practice will be found most certain, simple and safe, is to regard every case of neuralgia as having a dental origin, if there be any thing in the condition of the mouth which could produce it. In every case of neuralgia, especially those in which the head and face are the seat of disease, to examine *carefully* into the condition of the teeth and mouth. This examination, to be of any value, must be a thorough one. It is not enough to open the mouth and look into it, nor is it sufficient to "sound each tooth by striking it with a silver spoon," as recommeded by Dr. Darwin, though that is more than doctors have generally done.

The patient should be placed in a convenient chair, his head thrown back, so as to admit the light freely to every part of the mouth. The examiner should be provided with a suitable mirror, to reflect the posterior surfaces of the teeth, and a variety of small, variously curved instruments, to enable him to touch *every part of each tooth.* Then, beginning at some one place, he should take tooth by tooth, examining each one carefully in every part of it, and so pass round to the place of beginning.

His object in this examination will be:

1st. To find if in any tooth there be an exposed nerve.

2d. If any have lost their vitality, and have thus become a foreign substance in the mouth.

3d. If any be elongated or thrown partly out of its socket.

4th. If there be inflammation of the membrane lining the socket or covering the root.

5th. If there be alveolar abscess.

6th. If there be exostosis or bony enlargement of the fang.

7th. If any tooth be loose in its socket, from absorption of its alveolus.

8th. If there be any deposit of tartar or salivary calculi around the edges of the gum.

9th. If the gums are suppurating or inflamed.

10th. If any tooth be sensitive to the touch around its neck or on any part of its surface.

Such an examination cannot be made in a moment. It requires time, caution and skill. It should, if possible, be made by an educated and experienced dentist, who is familiar with the instrument to be employed, and with the ordinary appearances presented by the teeth and gums in health and in disease.

If all the parts thus examined, are found to be healthy, it will be rendered almost certain that the real seat of the neuralgia is not in the mouth, even though the teeth themselves should be the seat of pain.

Should one or more teeth be found diseased in some of the ways mentioned above, it would not follow of course that this is the cause of the neuralgia, but it would make it proper to adopt such treatment as would restore the mouth to health as a *probable* means of relief.

If, however, on touching any tooth, the pain is felt to extend at once to the seat of suffering. If the neuralgia be excited by striking any one with a steel instrument, or if the patient can distinctly trace the beginnings of the pain to any tooth, it would render it almost certain that the disease had a dental origin, and that it will only be permanently cured by the removal of its cause.

The only satisfactory evidence that it is *not* of dental origin would be the discovery of *some other evident and sufficient cause* or the restoration of the mouth to health without relief to the neuralgic suffering. The partial relief that is sometimes gained by the use of tonics, narcotics or other remedies addressed to the general system, is no evidence that the pain does not wholly or in part depend on the condition of the mouth, since I have often seen the most marked *temporary* benefit from such means when the event proved, beyond a doubt, that the case was of dental origin.

### *Treatment of Dental Neuralgia.*

The treatment of this affection may be gathered from what has been already said.

The great thing to be done is to get rid of the cause. *The mouth must be restored to health.* Every tooth that cannot be made healthy must be removed. Every dead tooth, every old root, and ordinarily, every loose tooth must be extracted. The gums must be restored. Every particle of tartar must be removed and proper powders and washes employed to secure the cleanliness of the teeth and the health of the gums. These things are essential. They must not be left undone. But in the meantime, I do not advise the neglect of all other means. The general condition of the health must be carefully regarded. If there be uterine, or bilious, or dyspeptic symptoms, they must be met by appropriate remedies. If the paroxysms return at regular intervals, much temporary advantage may be gained by the use of tonics, even though the true seat of irritation be in the mouth. In doubtful cases let every thing else be done which skill or science can suggest, but let not the restoration of the mouth be left undone.

*This can certainly do no harm.* The teeth which must be lost, are commonly of no value. The pain of extracting them is a mere trifle, compared with what the patient undergoes in every paroxysm; and even if the neuralgia be not relieved by their removal, the health of the mouth, the stomach, the lungs and the whole nervous system requires it. If it were an arm or a finger, or even a sound, healthy and useful tooth that must be sacrificed, the case would be different. But this is not needful. As a general thing, no teeth need be lost that ought not to be promptly removed, even if there were no neuralgia in the case.

Why then should the patient suffer month after month and year after year, spending hundreds of dollars with physicians and for nostrums, when a little temporary pain, sometimes the extraction of a single tooth, will effect not only temporary relief, but a perfect and permanent cure?

